# Medication Management in Portuguese Long-Term Care Facilities: A Preliminary Cross-Sectional Study

**DOI:** 10.3390/healthcare12212145

**Published:** 2024-10-29

**Authors:** Ana Rita Rodrigues, Filipa Mascarenhas-Melo, Victoria Bell

**Affiliations:** 1 Laboratory of Social Pharmacy and Public Health, Faculty of Pharmacy, University of Coimbra, 3000-548 Coimbra, Portugal; victoriabell@ff.uc.pt; 2 REQUIMTE/LAQV, Group of Pharmaceutical Technology, Faculty of Pharmacy, University of Coimbra, 3000-548 Coimbra, Portugal; filipamelo@ff.uc.pt; 3 Higher School of Health, Polytechnic Institute of Guarda, 6300-307 Guarda, Portugal

**Keywords:** ageing, long-term care facilities, medication management, consultant pharmacist

## Abstract

Background/Objectives: Population ageing has been a pressing global issue for decades. Older adults, especially those residing in long-term care facilities (LTCFs), often experience frailty and polypharmacy, which can lead to negative clinical outcomes. In Portugal, LTCFs provide essential care for individuals aged 65 or older, offering temporary or permanent accommodation. These facilities are not considered healthcare providers, and as a result, pharmaceutical services are not mandatory. This study aimed to evaluate medication management practices in Portuguese LTCFs, identify which professionals are responsible for managing medications, and identify potential gaps in safety and efficacy. Methods: A cross-sectional electronic questionnaire was sent by email to 2552 Portuguese LTCFs from the Portuguese “Carta Social” database. Data collection took place between 20 July and 2 August 2023, yielding a response rate of 15.4% (392 institutions). Results: Most LTCFs (94.39%) oversee their resident’s medication, with 75.95% using the same pharmacy. Individualised medication packaging is used by 57.84% of facilities, and 97.84% provide medication reconciliation and review, mainly conducted by physicians and nurses. Medication is often stored in nursing offices (81.12%) but also in kitchens or dining rooms. Nurses are responsible for medication storage (87.50%) and preparation (81.89%), although non-nursing staff are also involved. In 63.27% of LTCFs, the same individual is responsible for both the preparation and verification of medication. Assistants are involved in both the checking (30.56%) and administering (45.66%) of medication. Conclusions: The results presented illustrate the current status of LTCFs in Portugal. Medication management presents a significant challenge, and it is notable that the role of the pharmacist in this process is not as prominent as it is in other countries.

## 1. Introduction

Population ageing has been a long-standing global issue for several decades, particularly in Europe. This demographic shift is attributed to low fertility rates coupled with the increase in life expectancy. The population of older adults is defined as those aged 65 years or older, and it is estimated that it will reach 129.8 million by 2050 [1].

According to Eurostat, the average number of healthy life years at birth in the European Union in 2022 was 62.6 years. Conversely, in Portugal, the same metric was 59.1 years [2]. Regarding life expectancy at birth, the EU average in 2022 was 80.6 years, while in Portugal, this metric stood at 81.8 years [3]. This discrepancy between life expectancy and healthy life expectancy underscores the necessity of innovative solutions regarding health and social services for older adults. The healthcare system faces significant challenges due to these demographic changes, resulting in a greater demand for resources and healthcare professionals [4]. The increase in the proportion of the population that is older, the reduction in the number of years spent in good health and the fall in the birth rate present the Portuguese government with a number of new challenges [5].

Although ageing can have many meanings according to different societies [6], the World Health Organization (WHO) has established the concept of healthy ageing to ensure independence, create age-friendly environments at homes and among communities, align health and social systems and politics with the older adults’ needs, and to develop strategies for long-term care for older adults [7]. Population ageing is attributable to medical and social improvements [6], such as better nutrition, public health initiatives, and advancements in sanitation and hygiene [4]. However, it is also associated with multimorbidity and its concomitant costs [8]. It is expected that the incidence of falls, obesity, diabetes, and cardiovascular disease will continue to increase [4]. Studies have shown that the presence of multimorbidity is associated with polypharmacy, which can be a particular concern among long-term care facilities’ residents [9]. Polypharmacy is the term used to describe the consistent use of five or more medications [10]. According to Allin et al., polypharmacy is a multifactorial condition, reflecting older adults’ comorbidities, the availability of drug treatments, and the clinical guidelines that recommend multiple medications to treat a single condition [11]. Patients undergoing polypharmacy are at risk due to prolonged use, incorrect dosing, inefficacy, outdated medications, drug–drug interactions, and interactions with comorbidities. These risks are collectively referred to as potential inappropriate medication (PIM) [10]. PIM is used to describe medication that should not be prescribed to older adults because the risk of adverse events outweighs the clinical benefits [12]. The risk of adverse events is even greater for older adults because of decreased renal and hepatic function, lower lean body mass, and reduced hearing, vision, cognition, and mobility [13]. Polypharmacy has been linked to several negative health outcomes, such as frailty, hospitalisation, and mortality, and some studies have also shown an association between polypharmacy and falls, cognitive impairment, and physical function [14].

There are some tools to evaluate if a medication is inappropriate for an older adult, such as the Beers Criteria [15] and STOPP/START Criteria [16]. Benzodiazepines are the most common PIM [17] and are associated with cognitive impairment, falls, and fractures [12]. Other reported PIMs are non-steroidal anti-inflammatory drugs, antidepressants, and antihistamines [18,19].

Medication discrepancies may be more prevalent among older adults with multiple prescribers, frequent transitions between care providers, and a reduced ability to recall their current medication regimens [20]. Medication management is a set of strategies that aid individuals in adhering to their medication. According to Liau et al. [20], medication management recalls in seven principles: perform medication reconciliation, assess and plan based on the capacity of self-management of medications, appropriate prescribing and deprescribing, simplify medication regimens, be alert to medications contributing to geriatric syndromes, review medication regimens regularly, and facilitate multidisciplinary communications.

Medication management is defined as a patient-centred approach to optimising medication use and enhancing patient health outcomes [21]. It is a multifaceted process and comprises various stages: (1) ordering, where the physician prescribes the medication [22,23,24]; (2) transcribing, when the pharmacist enters the order into the pharmacy computer system or the nurse hand copied into the medication administration record [22,24]; (3) preparing the medication by the pharmacist or pharmacy technician [23,24]; (4) dispensing, by the pharmacist [22,23,24]; (5) administering the medication by the nurse [23,24]; and (6) monitoring and reporting by all healthcare team members [23,24]. A medication error may occur at any of stage of the medication-use process [25].

A qualitative observational study conducted by Odberg et al. [22] analysed medication administration in two Norwegian LTCFs and identified it as a complex process requiring interprofessional collaboration. The procedures involved vary depending on the technological solutions, documentation requirements, and staff discretion in performing medication-related tasks. LTCFs have a higher number of residents than hospital patients [26,27,28]. However, the use of health systems to record medication administration electronically is not yet mandatory in these facilities [25]. A study by Szczepura et al. [26] revealed that 90% of LTCF residents were exposed to at least one potential administration error. Nevertheless, minimal attention has been given to LTCF residents regarding medical errors and their prevention [29]. Although some studies report the incidence of adverse drug events in LTCF worldwide [30] and the benefits regarding pharmacist intervention [31,32], studies reporting on Portugal are scarce.

Pharmacist-led interventions in medication management are well-documented in the literature and have been proven effective in reducing medication-related problems, increasing the quality of medication regimens, and improving medical outcomes [33,34]. It includes medication reconciliation, medication review, and deprescribing.

Medication reconciliation is the procedure where the most accurate list of all medication a patient is taking is created at transition points in the health system. This step improves patient safety, and hence, omissions, unnecessary duplicate therapies, or the incorrect dosing of medications are avoided [35]. According to a study from Elliott et al., medication discrepancies frequently occur during transitions from hospital to long-term care, with 20% of residents experiencing medication errors within 24 h of being discharged [36]. Older adults are likely to benefit from medication reconciliation due to high rates of polypharmacy and complex medication regimens.

Medication review is a comprehensive and structured evaluation of all medicines taken by a patient, highlighting actual or potential pharmacotherapy problems, and providing recommendations to solve them [30]. Some identified problems are “inappropriate dose”, “inadequate dosage”, “medicine use without indication or unclear indication”, “cheaper alternative”, “drug-drug interaction”, “untreated condition”, or “no monitoring” [37,38]. Pharmacists can recommend dose adjustment, deprescription, the ceasing of medication, alternative medication, such as cheaper alternatives with equal effectiveness, an alternative dose form, monitoring or investigative tests, and others [39]. Since older adults tend to be prescribed more medicines, it is expected that this population will benefit most from this kind of service [30].

Deprescribing is the process of the withdrawal of inappropriate medication in order to manage polypharmacy. A recent meta-analysis concluded that deprescribing in LTCF reduces the odds of PIM by 59% [40]. This pharmacist-led intervention can also impact other health outcomes, such as reducing costs, improving medication adherence, lowering the incidence of polypharmacy, and reducing the pill burden of LTCFs’ residents [34]. Opioids and benzodiazepines are commonly used in older adults. They are often used to treat conditions that have a significant impact on patients’ quality of life, such as pain, anxiety, and insomnia. However, these medications can lead to falls and other serious adverse effects in older adults [41]. As a result, health professionals have been working to decrease their use.

In some countries, such as the USA, legislation already mandates the conducting of monthly medication reviews [31]. Furthermore, it is predicted that a Consultant Pharmacist must monitor medication regimens in LTCFs [42,43]. Medicare is an American insurance programme for individuals aged 65 or above or those with disabilities [44]. Since 2013, the programme has included an annual medication therapy management service, which comprises a comprehensive review of medication [45] for all beneficiaries, including residents in LTCFs [46].

In Canada, primary care is included in the country’s universal health coverage system, which includes pharmacists. Nevertheless, in Ontario and Quebec, individuals aged 65 or above are eligible for public drug coverage [11]. In Ontario, the review of the medication is a remunerated service. MedsCheck, which was introduced in 2006, is a publicly funded community pharmacy medication review service for LTCF residents and people taking at least three prescription medications for a chronic condition [11,47].

In Slovenia, the Slovenian National Health Insurance Institute has included a medication review service since 2012, with the objective of more effectively addressing the issue of polypharmacy. This project follows the Netherlands model, in which the pharmacist works within a primary care multidisciplinary team comprising 8-12 professionals [48]. In this system, General Practitioners (GPs) are able to refer patients to the clinical pharmacist for a medication review. The Consultant Pharmacist conducts an optional patient interview and performs a medication review to assess potential drug–drug interactions, possible adverse events, potential prescribing omissions, potentially inappropriate medications, and medication adherence. In their report, sent to the GP, they include their recommendations. The programme has been the subject of reimbursement by the Slovenian government since 2016.

In the United Kingdom, all community pharmacists are contracted by the NHS to provide pharmaceutical services, such as dispensing medication and medicine use reviews [49]. These services are reimbursed by the NHS and free of cost for patients.

Health systems worldwide are facing significant pressures from various challenges, including ageing, an increase in chronic conditions, and a forecasted global shortage of medical doctors [50]. Pharmacist-independent prescribers started in the UK in order to meet the increased health care demand, and they have been expanding to Canada, New Zealand, and the USA [51]. Pharmacists can register as independent prescribers and prescribe any medicine for any medical condition within their areas of competency [52]. Some studies show comparable outcomes for some parameters, such as systolic blood pressure, medication adherence, and patient satisfaction, in non-medical prescribers regarding the management of chronic conditions [53]. Pharmacist-independent prescribers can reduce the time physicians spend on prescribing activities, reducing their workload, and increasing patient access to healthcare [54]. As independent prescribers, pharmacists are responsible for patient care and clinical assessments. They also handle the initiation of therapy and follow-up of treatment [55]. Thus, they are responsible for nearly the entire process of medication management.

Worldwide, pharmacists have been gathering new responsibilities in non-hospital settings [32]. These include providing consultation for dwelling cancer patients [56,57], participating in prescribing [51], and deprescribing [34], and assisting patients with medication management [58].

This study aims to enhance the understanding of medication management in Portuguese LTCFs, where pharmacists are not mandatory staff members. This research will evaluate the current methodologies employed for managing medications in these settings and identify potential gaps in safety and efficacy. The study examines the existing practices and responsibilities of different professionals in LTCFs’ with regard to medication management.

## 2. Materials and Methods

This study had a cross-sectional design, using an electronic questionnaire distributed to Portuguese long-term care facilities for older adults. The questionnaire was designed to collect demographic data and to assess medication management practices.

### 2.1. Study Design

This was a cross-sectional study. It used an electronic questionnaire for data collection and distributed it via e-mail to Portuguese long-term care facilities for older adults. The e-mail addresses were sourced from the Portuguese “Carta Social” database [28]. To guarantee that the questionnaire was intelligible, clearly delineated, and effectively aligned with the objectives of the study, two experts from the related field evaluated its face validity. Participants were informed that anonymity and confidentiality were ensured. This study is not subject to ethics committee validation as it focuses on institutions rather than on people or animals. It contained two main sections:

**Section 1:** Demographic data collection, including location, staff, and capacity;

**Section 2:** Medication Management, which was divided into four approaches: Acquisition (seven questions); Storage (six questions); Preparation (four questions); and Administration (two questions).

The questionnaire was developed using Microsoft Office forms (Edition Office 356 A3 for students). All questions were multiple choice in order to reduce the probability of keystroke errors and facilitate data analyses. All answers were mandatory, and the technology only allowed for the submission of fully completed fields, to eliminate missing data.

### 2.2. Setting/Context

**Figure 1:**
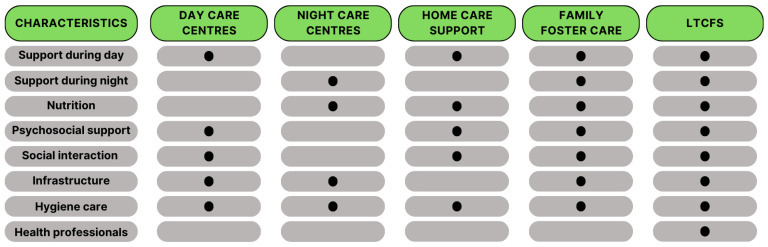
Portuguese social mechanisms to support older adults and their characteristics (indicated by dots).

In Portugal, there are five mechanisms to support older adults [59]: day care centres [60], night care centres [61], home care support services [62], family foster care for older adults [63], and long-term care facilities [64,65]. These services are described in Figure 1.

Long-term care facilities are a formal care system for older adults, providing various professional services [66]. These facilities are social institutions intended for people aged 65 or older, providing temporary and permanent accommodation. They deliver a wide range of social support activities and care to meet the specific needs of residents and their families [64], facilitating independent and safe living while performing daily activities that would be challenging or impossible if living alone [66].

The primary goals of LTCFs include providing consistent and appropriate care, supporting the healthy ageing process, and enhancing social inclusion while upholding the rights, liberties, and guarantees of residents. The main reason for using an LTCF is disability [66]. To meet the needs of their residents, LTCFs must provide services, such as adequate nutrition, hygiene and comfort care, cultural, social, and ludic activities, assistance with daily tasks, nursing care, access to healthcare, and medication administration. Besides those, LTCFs can also provide other care services to ameliorate the quality of life of its residents [64].

Portuguese LTCFs are obliged to ensure appropriate staffing, which must include a technical director, a sociocultural animator, social educator, or geriatrics technician, a nurse, an assistant, a person responsible for domestic services, a cook, a cook’s assistant, and an auxiliary employee [64]. Since LTCFs can provide other services, nutritionists, physiotherapists, and physicians can also be employed. The staffing levels are determined by the number of residents. However, in institutions with fewer than 20 residents, it is only mandatory to have a technical director, an assistant, and an auxiliary employee.

The questionnaires were sent between 20 July and 2 August 2023, to 2552 Portuguese long-term care facilities. In the districts with the lowest response rates, telephone calls were made to alert people to the email questionnaire and to encourage institutions to respond. Only responses received by 31 March 2024, were included. All responses were carefully analysed and deemed valid for inclusion in the study.

#### 2.2.1. Participants

The inclusion criteria were legal Portuguese LTCFs, registered with the Social Security system and listed on the Portuguese government’s “Carta Social” platform. All LTCFs were included in the study, regardless of their private, social/solidarity, or other nature.

#### 2.2.2. Sampling Method and Sample Size

In Portugal, as of 2022, there were 2552 LTCFs with a total capacity of 103,420 residents [28]. In terms of ownership, LTCFs in Portugal can be either private or social (Private Institutions of Social Solidarity or Holy House of Mercy). A total of 392 responses were obtained, resulting in a final sample size of 392 institutions (*n = 392*) institutions. This represents 15.4% of the total number of facilities in the country. In order to account for a margin of error of 5% and a 95% confidence level, the following equation was applied: Necessary Sample Size = ((Z-score)^2^ × StdDev × (1-StdDev))/(margin of error)^2^. The minimum sample size was 335. The data were analysed using Microsoft Office Excel (Edition Office 356 A3 for students).

## 3. Results

**Figure 2:**
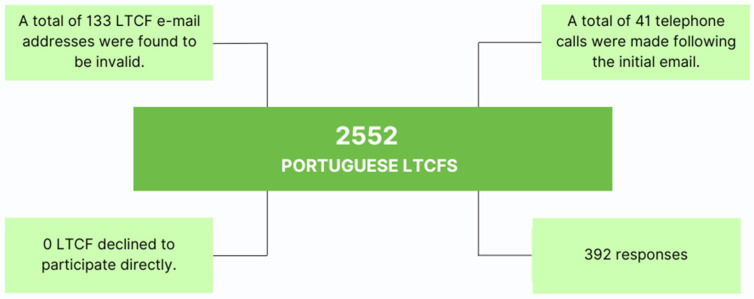
Diagram of LTCF recruitment.

Of the 2552 Portuguese LTCFs registered in “Carta Social”, a total of 392 institutions participated in the study. Figure 2 shows the flow diagram of LTCFs’ contact to recruit the institutions to the study.

### 3.1. General Data on LTCFs

Table 1 presents a summary of the respondents’ demographic data and its comparison to the national data available in the “Carta Social” website (data from 2022). The study encompasses all districts of Portugal, and the distribution of responses is in close alignment with the national weighting. Table 1 presents a comparison between the obtained responses and the national data, including the percentage weighting of each district. In terms of bed capacity, 206 LTCFs (52.55%) have between 20 and 49 beds, indicating a comparable level of accommodation for residents. The mean number of beds among the participating LTCFs is 43.01, while the national average is 40.53. Most institutions belong to the social sector, namely Private Institutions of Social Solidarity and Holy House of Mercy—290 (73.98%) of the participating institutions. Additionally, 98 (25%) belong to the private for-profit sector. The data from the LTCFs in this study are consistent with the national data reported in the “Carta Social”.

Table 2 presents the professional categories employed in all respondent’s LTCFs. All facilities could mark required professions and list any additional staff members. The majority of institutions employ professionals who are legally required to be present, and many also employ other professionals whose role is to provide different care and help with the institutions’ legal obligations.

### 3.2. Medication Management Results

Te questionnaire included four subsections regarding medication management, each about a stage in the medication circuit: acquisition, storage, medication preparation, and administration to residents. The data are organised according to each subsection.

#### 3.2.1. Acquisition results

The aim of “Acquisition” was to ascertain which individual or entity is responsible for acquiring medications for residents. The results are presented in Table 3. Most LTCFs—206 (52.55%)—indicate that they are responsible for purchasing the medications for their residents. In 164 (41.84%), the responsibility is shared between the institution and the residents or their families. Only 22 respondents (5.61%) stated that acquiring medications is solely the responsibility of the residents and their families. As the study focuses on medication management in LTCFs, only responses indicating that the acquisition responsibility is entirely or partially on the LTCF were considered in the subsequent questions of this section.

Regarding where medications are acquired, 281 (75.95%) LTCFs consistently use the same pharmacy, while 89 (24.05%) use a different one.

A total of 214 (57.84%) LTCFs confirmed that pharmacies prepare medications individually for each resident using specific devices, while 156 (42.16%) reported that they do not provide this service. Lastly, LTCFs were asked about the availability of medication review and reconciliation services for residents; 362 (97.84%) report that these services are available to their residents, while 8 (2.16%) do not. In 348 (96.13%) of the cases, the LTCF is responsible for these services, unlike 14 (3.87%) facilities where this responsibility relies on the community pharmacy (Table 4).

**Figure 3:**
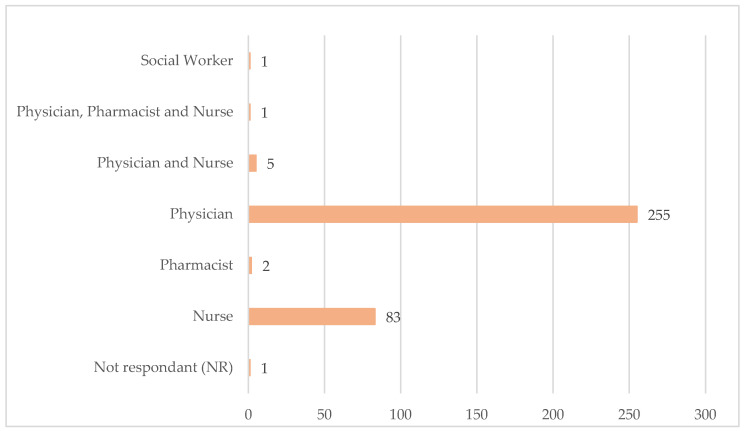
Professionals performing review and reconciliation of medications.

Of the 348 institutions that provide medication review and reconciliation services, 260 employ the services of a physician, either individually or in collaboration with other professionals (nurses), 2 institutions employ the services of a pharmacist, and 83 employ the services of a nurse. In one institution, a healthcare team comprising a physician, nurse, and pharmacist is responsible for this service. In another, a social worker assumes this responsibility (Figure 3).

#### 3.2.2. Storage results

Regarding the storage of medications in LTCFs, we first asked about the internal procedures for checking expiration dates and ensuring proper storage and preservation conditions, such as maintaining an appropriate temperature, humidity levels, and security measures. As shown in Table 5, 288 (73.47%) LTCFs report that these responsibilities fall on the institution itself, while 104 (26.53%) indicate that the pharmacy is responsible for these tasks.

The storage locations for different types of medications were then evaluated, focusing on those without special storage conditions, those requiring refrigeration between 2 °C and 8 °C, and medications containing psychoactive or narcotic substances, as outlined in Table 6.

Most LTCFs store all three types of medications in nursing offices: 313 (79.85%) LTCFs for medications without special storage requirements, 311 (79.34%) for refrigerated medications, and 330 (84.18%) for psychoactive or narcotic medications.

A smaller percentage of LTCFs have a designated area for storing medications, referred to as the internal “Pharmacy”. Here, 41 (10.46%) LTCFs store medications without special requirements, 25 (6.38%) store refrigerated medications, and 28 (7.14%) store psychoactive or narcotic medications. Some facilities report a medication cabinet for medication storage. Nine (2.30%) LTCFs store medications without special storage requirements, and 23 (5.87%) store psychoactive or narcotic medications in this location.

Some LTCFs use other areas within the facility for storage, but these are less common. The dining area is used in 6 (1.53%) institutions for storing medications without special storage requirements, 17 (4.34%) for refrigerated medications, and 2 (0.51%) for psychoactive or narcotic medications. Additionally, 11 (2.81%) and 8 (2.04%) facilities store refrigerated medications in the kitchen or kitchen pantry.

One LTCF (0.26%) stores all medications in the office, two (0.51%) store the medication in the resident’s room, and some facilities use a support room, with one (0.26%) for medication without special requirements, nine (2.30%) for refrigerated medications, and two (0.51%) for psychoactive and narcotic medications.

Seven (1.79%) facilities use medication carts for storing medications.

**Figure 4:**
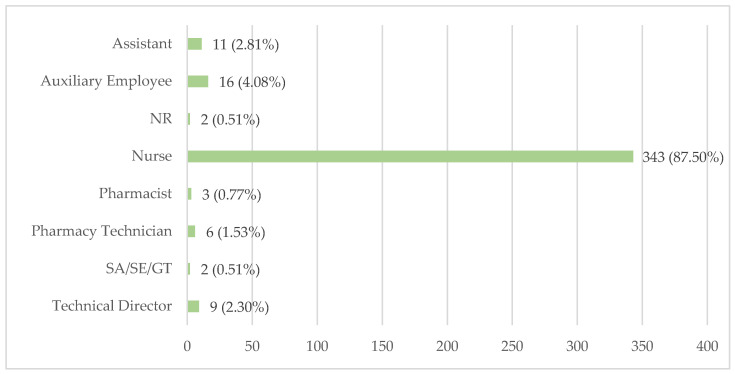
Professionals responsible for medication storage.

The LTCFs were asked to identify the professional category responsible for medication storage, and the results are shown in Figure 4. Nurses are most commonly responsible for storing residents’ medications, having this duty in 343 LTCFs (87.50%). Following nurses, auxiliary employees are responsible for storage in 16 LTCFs (4.08%, with assistants in 11 LTCFs (2.81%).

In some institutions, the responsibility is assigned to the Technical Director, which occurs in nine LTCFs (2.30%), or to pharmacy technicians in six LTCFs (1.53%). Pharmacists are responsible for medication storage in only three LTCFs (0.77%). Additionally, two facilities (0.51%) assign this responsibility to a sociocultural animator/social educator/geriatrics technician (SA/SE/GT).

#### 3.2.3. Preparation and Verification results

**Figure 5:**
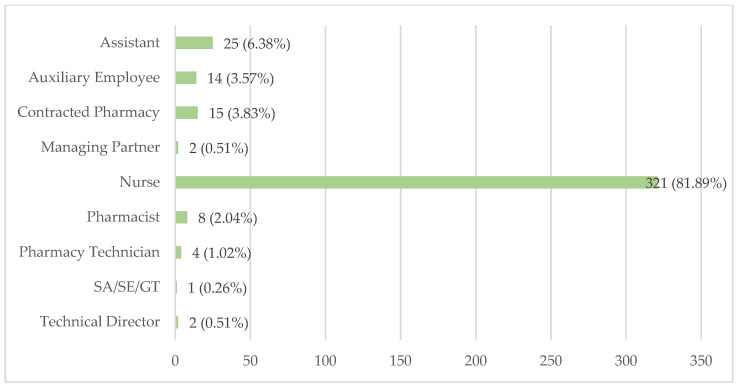
Professionals responsible for medication preparation.

The LTCFs identified which professionals are responsible for medication preparation, as shown in Figure 5.

Most LTCFs assign this task to nurses, who are responsible in 321 institutions (81.89%). Assistants handle medication preparation in 25 LTCFs (6.38%), while auxiliary employees are responsible in 14 (3.57%).

Other professionals also participate in medication preparation, though to a lesser extent. Managing partners and technical directors each account for two facilities (0.51%), and SA/SE/GT are responsible in one LTCF (0.26%).

In the pharmaceutical sector, 15 LTCFs (3.83%) rely on a contracted pharmacy to prepare all medications. Pharmacists are responsible for medication preparation in eight LTCFs (2.04%), and pharmacy technicians are responsible in four (1.02%).

The involvement of other professionals in medication preparation includes assistants in 25 LTCFs (6.38%), auxiliary employees in 14 (3.57%), managing partners and technical directors in 2 institutions each (0.51%), and SA/SE/GT in 1 (0.26%).

After inquiring about medication preparation, we asked LTCFs about their verification process; 248 LTCFs (63.27%) report that the verification of medication preparation is carried out by the same professional who prepared the medication. In contrast, only 144 facilities (36.73%) have a different staff member handle the verification (Table 7).

**Figure 6:**
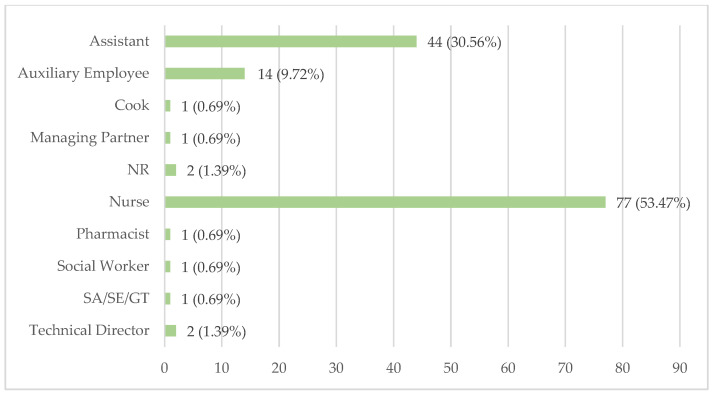
Professionals responsible for medication preparation verification.

For LTCFs where medication preparation and verification are handled by different professionals, they were asked about the category of the individual responsible for the verification. The data on these professional categories are shown in Figure 6.

Nurses are most frequently responsible for medication preparation verification, accounting for 77 LTCFs (53.47%). Assistants follow, taking on this responsibility in 44 facilities (30.56%), while auxiliary employees are involved in 14 (9.72%).

Technical directors are responsible for verification in two facilities (1.39%). Additionally, in one LTCF (0.69%), this responsibility is assigned to an SA/SE/GT, social workers, pharmacists, managing partners, and cooks, each.

#### 3.2.4. Administration results

**Figure 7:**
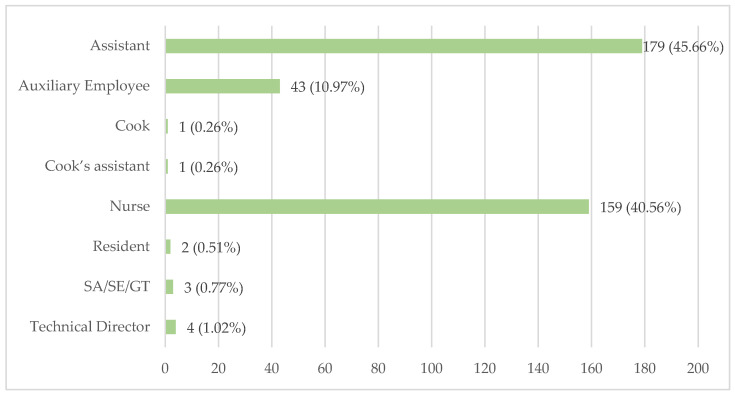
Professionals responsible for medication administration.

LTCFs also provided information on which professionals are responsible for administering medications to residents. The data on these categories can be seen in Figure 7.

Assistants are the largest group involved in medication administration, accounting for 179 LTCFs (45.66%), followed by nurses, who assume this responsibility in 159 institutions (40.56%).

Auxiliary employees are responsible for medication administration in 43 LTCFs (10.97%). Other staff members assigned this duty include the technical director, in 4 facilities (1.02%), SA/SE/GT in 3 (0.77%), residents themselves in 2 (0.51%), and both the cook and cook’s assistant in 1 LTCF (0.26%) each.

## 4. Discussion

Older adults, particularly those who live in LTCFs, are generally frailer, have more chronic conditions, and are polymedicated [9,18,67]. In Europe, about 26% of community-dwelling patients and 43% of nursing home residents are exposed to PIM [47,67,68], which is a significant risk factor for adverse drug events [69] and poor outcomes [70]. PIM is related to a poor quality of life, high morbidity, preventable adverse drug events, an increased risk for falls, and repeated hospitalisations [10,33]. PIM can also have implications for the avoidable health system cost [11,67]. In addition, medication-related symptoms tend to be difficult to distinguish from symptoms related to older adults’ comorbidities [43]. Hence, medication management in older adults in long-term care facilities is crucial to prevent unnecessary harm [9].

Nurses assume a significant role managing medication in Portuguese long-term care facilities (LTCFs). They are responsible for the storage (87.50%), preparation (81.89%), validation (53.47%), and administration (40.56%) of residents’ medications.

This study examined how staff in LTCFs manage medication and aimed to provide a better understanding of the role of pharmacists in LTCFs.

### 4.1. Medication ManagementDiscussion

#### 4.1.1. Acquisition Discussion

The data regarding acquisition provides valuable insights into the medication management practices of LTCFs. It is noteworthy that only 22 residents (5.61%) and their families are solely responsible for medication acquisition, while 370 (94.39%) rely entirely or partially on the LTCF for this task. Additionally, it is interesting to observe these data, when considered alongside the observation that 281 facilities (75.95%) consistently purchase medications from the same pharmacy, despite the fact that according to Portuguese Decree-Law 307/2007 [71], users have the right to freely choose their pharmacy. This suggests that the freedom of choice in pharmacy selection may not be fully realised in practice.

Pharmaceutical services, such as drug dispensation, medication review and reconciliation, and Individualized Patient Medication Packaging, play a crucial role in promoting the health and well-being of older adults by enhancing therapy adherence and improving their quality of life [72].

Notably, 362 LTCFs (97.84%) indicated that their residents had access to medication review and reconciliation services. Surprisingly, 348 facilities (96.13%) mentioned that these services were provided within the LTCFs themselves. However, it is important to consider that according to Directive 018/2016 from the Portuguese Directorate-General of Health [73], medication reconciliation services should be carried out by a healthcare professional.

Although 13 institutions reported having a pharmacist on staff, only 3 of these institutions have pharmacists providing medication review and reconciliation services to the LTCFs’ residents. Nurses are also heavily involved in medication review and reconciliation, which is expected, as having a nurse on staff is a mandatory requirement for Portuguese LTCFs. However, in one institution, this service is performed by a social worker, a professional who lacks the healthcare training necessary to fulfil this role. Studies concerning patient safety in long-term care institutions have concluded that insufficient medical staff, scarce facilities, and underfunding can increase the risk of adverse events [74]. Approximately 70% of adverse events can be prevented [75]. Increasing medication safety in LTCFs can improve health outcomes and reduce the healthcare costs associated with these errors [76]. A systematic review and meta-analysis by Lee et al. [9] analysed pharmacist services in LTCF, and found that clinical medication reviews, including medication reconciliation, were the central activities performed in these institutions. This study also identifies other initiatives where pharmacists can be involved, such as participating in multidisciplinary LTCF team meetings and staff education on topics, such as falls or stroke prevention. The review reveals that pharmacist intervention has reduced the mean number of falls among residents and reduced mortality rates, hospitalisation, and admission rates, albeit not significantly. It also identified that pharmacist intervention has led to an improvement in the quality of prescribing and reduced the number of medicines taken by residents. Considering these results, the study concludes that LTCF healthcare teams should include pharmacists.

#### 4.1.2. Storage Discussion

The investigation initially focused on ascertaining the internal procedures employed by LTCFs to verify medication expiration dates and ensure proper storage, regarding temperature, humidity, and security. In Portugal, LTCFs are the responsibility of the Ministry of Labour, Solidarity, and Social Security and its Social Security Services [77] and are not considered healthcare providers. For this reason, pharmaceutical services are not mandatory in these institutions. Portugal has a National Network for Long-Term Integrated Care (LTICN) [78] that provides healthcare for those discharged from hospital settings but who still need care from health professionals. This network is a shared responsibility between the Ministry of Health and the Ministry of Labour, Solidarity, and Social Security [79]. The LTCIN comprises all forms of continuous, rehabilitation, palliative, and nursing care for people with limitations.

Unlike healthcare institutions, such as those in the integrated care network [78], hospital pharmacies [80], community pharmacies [81], and pharmaceutical distribution [82], LTCFs, being considered social facilities [64] rather than healthcare institutions, do not have explicit legal regulations outlining rules for medication storage and preservation.

The questionnaire revealed that in 288 LTCFs (73.47%), the responsibility for verifying medication expiration dates and ensuring proper storage and preservation conditions lies with the LTCF itself, while in 104 (26.53%), this responsibility is assigned to the pharmacy. Notably, according to best practices in community pharmacy [81], pharmacies can only regulate storage conditions within their premises, making it impossible to oversee once the medication has left the pharmacy circuit. It is also noteworthy that none of the institutions indicated that the responsibility for medication storage falls on the residents themselves, signifying that the LTCF indeed assumes control over managing their residents’ medications.

In line with best practices in community pharmacy [81], hospital pharmacy [80], and pharmaceutical distribution [82], medications must be stored under appropriate temperature, light, and humidity conditions. However, the absence of specific legal provisions regarding medication storage in LTCFs means that Portuguese law does not outline any explicit rules in this regard.

The majority of LTCFs store residents’ medications in the nursing office, which is the mandatory health area described in the legislation [64], encompassing 313 (79.85%) LTCFs for medications without special storage requirements, 311 (79.34%) for refrigerated medications, and 330 (84.18%) for psychoactive or narcotic medications.

Some LTCFs have recognised the importance of having dedicated spaces for medication storage, such as internal “pharmacies”, designated rooms for medication storage (41 (10.46%) LTCFs store medications without special requirements, 25 (6.38%) store refrigerated medications, and 28 (7.14%) store psychoactive or narcotic medications in this area), medication carts, similar to those used in hospital settings, and medication cabinets.

Conversely, certain LTCFs utilise less suitable areas for medication storage, such as the dining area, kitchen, pantry, recreation room, and activity room, posing risks to appropriate storage conditions and increasing the likelihood of medication errors, such as mix-ups between residents’ medications. While their utilisation is constrained, there is a potential to compromise the safety of the residents, which may result in errors, the inadvertent administration of medications, and a decline in the quality of medications due to inadequate storage conditions. The primary responsibility for medication storage lies with nurses, as 343 LTCFs (87.50%) assign this duty to them. However, this places significant pressure on the nursing staff, who already have various clinical and administrative responsibilities. Involvement in medication storage is also observed among auxiliary employees, responsible in 16 (4.08%) LTCFs, assistants, responsible in 11 (2.81%), the technical director, responsible in 9 LTCFs (2.30%), pharmacy technician, responsible in 6 LTCFs (1.53%), and an SA/SE/GT, responsible for storage in 2 facilities (0.51%). These data raise concerns about the adequacy of their training and expertise in medication handling, which could impact the quality and safety of medication storage practices.

Surprisingly, only three institutions (0.77%) assign the medication storage responsibility to pharmacists. Increasing the involvement of pharmacists could potentially enhance the accuracy and safety of medication management practices within LTCFs.

Additionally, the emergence of pharmacy technicians in six LTCFs (1.53%) as professionals responsible for medication storage is an interesting finding, potentially attributable to diverse categorisations of staff members or outsourced services.

#### 4.1.3. Preparation and Verification Discussion

A study from Co-LABOR (Collaborative Laboratory for Work, Employment and Social Protection) [83] evaluated the impact of COVID-19 on Portuguese LTCFs and concluded that 7.3% of the residents in these institutions did not have a family physician, and 52.8% were not followed in the primary care settings despite their morbidity and health status. This study also highlighted that the current regulatory framework is insufficient and suggested that it should be revised. The research team also emphasised that further studies are necessary to characterise LTCF residents and to identify health-related problems.

In accordance with good practices for healthcare provision in LTCFs, overseen by the General Inspectorate of Health Activities [84], medication preparation for LTCF residents should be carried out by nurses if pharmaceutical services are not involved. The results indicate that this scenario is common in most LTCFs in Portugal. Moreover, there is a notable preference for nurses to handle medication preparation over pharmaceutical services, such as pharmacists or contracted pharmacies.

Three-hundred and twenty-one LTCFs (81.89%) rely on nurses for medication preparation. This high percentage aligns with best practices, given that nurses are the only mandatory health professional in Portuguese LTCFs. However, we cannot overlook the presence of professionals without formal healthcare training who are responsible for medication preparation, such as assistants in 25 LTCFs (6.38%), auxiliary employees in 14 (3.57%), and an SA/SE/GT in 1 (0.51%). This assignment could compromise resident safety and does not align with current standards and recommendations. Only 15 LTCFs (3.83%) report that medication preparation is handled by contracted pharmacies, which contradicts the data from the acquisition subsection regarding Individualized Patient Medication Packaging, where 214 LTCFs (57.84%) indicated that this service was provided directly by the pharmacy. Further studies are needed to better understand this discrepancy. Once again, pharmacists are responsible for preparation in only four LTCFs (2.04%), which could be higher given their expertise in this field.

It was found that 248 LTCFs (63.27%) have the same individual responsible for both preparing and verifying medication. This raises concerns about potential oversight and errors, as there is a lack of independent checks to catch mistakes and ensure accuracy. A study from Dilles et al. [85] identified a lack of time to double-check medication before administration as one barrier to safe medication management in LTCFs. The risk of unnoticed medication errors is increased, especially if the individual is under time pressure or if errors are subtle. In contrast, 144 facilities (36.73%) have medication verification performed by a different staff member, which is crucial for enhancing medication safety. An independent verification provides an additional layer of scrutiny, helping to identify errors or discrepancies that the original preparer might have missed. This practice aligns with the best practices in medication management.

Nurses are the predominant professionals responsible for verifying prepared medication in 77 LTCFs (53.47%). However, this percentage is lower compared to their responsibility in other medication management tasks. This discrepancy may be due to LTCFs wanting verification to be performed by professionals different from those who prepared the medication. As a result, assistants take on more responsibility for verification with 44 LTCFs (30.56%), as well as auxiliary employees in 14 (9.72%). Other professionals have minimal representation in the responsibility of verification. In particular, the appearance of cooks in medication management, who are not adequately trained for this task, is surprising. Best practice guidelines indicate that the verification of medication administration should be performed by a nurse [84], which is not being followed according to these data.

Pharmacist-led medication management can improve therapy adherence and health literacy and decrease patient out-of-pocket costs [86,87]. Additionally, lower out-of-pocket costs may promote medication adherence, especially among lower-income beneficiaries [86]. Consultant Pharmacists can provide strategies for decreasing the dosing frequency, by combining similar dosing-frequency medication to be dosed at the same time or by switching to longer-acting formulations, reducing the pill burden, delivering special administration instructions regarding dosage forms, and other medication-related characteristics [42,43]. Consultant Pharmacists can also track the progress of the therapeutic goals and update the physician on any challenges in achieving them [43]. This can lead to better identification of medication-related problems or treatment failures.

A pharmacist care programme implemented in Hawaii, including hospital and community pharmacists, showed a 36% reduction in the rate of medication-related hospitalisations and estimated savings of 6.6 million dollars per year in avoided hospitalisations [88]. In some countries, such as Sweden, the United Kingdom (UK), the United States of America (USA), Switzerland, or Australia, pharmacist-led intervention is already part of the health system. However, most studies focus on community intervention instead of aged care institutions, such as LTCFs [9]. Another study by Entsuah et al. [89] evaluated consultant pharmacist recommendations in 24 LTCFs, with 603 residents. This study found that half of the patients required one or more recommendations, mainly concerning medication monitoring, medication without indication, and overdosage, and 2% of the recommendations were concerning medication errors.

Medication-related problems are particularly prevalent in LTCFs, especially the prescription of PIM, with some studies concluding that 15 to 50% of residents were subjected to PIM [40]. Moreover, inappropriate medication is associated with higher hospitalisation rates and mortality in older patients [35]. Pharmacist-led interventions regarding medication use can optimize prescribing practices and improve LTCFs’ residents’ health outcomes, and consultant pharmacists may have a pivotal role [23].

#### 4.1.4. Administration Discussion

The information provided in Figure 7 outlines the different professional categories involved in the administration of medications. Understanding these roles is essential for evaluating whether medication administration aligns with best practices and regulatory standards. Administering medications requires in-depth knowledge of drug interactions, accurate dosages, and potential side effects. Professionals without formal training in these areas may lack the expertise needed to handle these tasks safely and effectively, which could result in medication errors or adverse outcomes for residents.

Assistants make up the largest group involved in medication administration, being responsible in 179 LTCFs (45.66%). Their significant involvement highlights the reliance on these staff members, whose training may vary widely, raising potential concerns about the thoroughness and safety of the process. In accordance with best practice guidelines [84] and professional practice regulations, nurses are typically responsible for medication administration, which is not the case in the inquired LTCF. The inclusion of other roles such as an SA/SE/GT in three LTCFs (0.77%) and cooks and cook’s assistants in one (0.26%) LTCF each is particularly concerning due to their lack of appropriate medical training. Additionally, the dosing and administering of medications are vulnerable stages at which adverse events are more likely to occur [22].

This diverse approach indicates potential risks to medication safety, highlighting the need for further investigation into medication management in LTCFs and a re-evaluation of role assignments to prioritise clinically trained professionals and robust oversight mechanisms. Addressing these issues can enhance the safety and effectiveness of medication management processes in LTCFs, ultimately leading to better outcomes for residents.

In LTCFs, there are interventions that can increase patient safety, such as electronic medical records to lower the number of medical errors and having a pharmacist coordinating medication verification and administration to reduce hospitalisations and mortality [74].

### 4.2. Strengths, Weaknesses, and Limitations

To the best of our knowledge, this is the inaugural cross-sectional study to elucidate medication management practices in Portuguese LTCFs. Previously published studies have only involved a limited number of subjects from specific regions or have focused on a review of Portuguese legal requirements. The present study, which employed a questionnaire, represents the most comprehensive investigation to date on medication management in LTCFs, encompassing representative institutions from across the entire country.

Although the results are statistically significant, it would be beneficial to include more institutions, representing all Portuguese municipalities, in order to obtain a more comprehensive understanding of the pharmaceutical management practices in LTCFs in Portugal. To this end, it may be necessary to emphasise to participants the necessity of responding to the questionnaire, rather than merely using the email. The present study is potentially limited by the fact that the questionnaire was validated by only two specialists in the field of sociopharmacy. It might have been beneficial to have the questionnaire validated by professionals from LTCFs as well, in order to reduce the likelihood of misinterpretation of the questions. It would be beneficial for future research to confirm these findings using an alternative questionnaire.

### 4.3. Future Perspectives

Given the challenges in medication management in LTCFs, especially when only nurses are the mandatory healthcare professionals on the team, a single individual cannot effectively manage all tasks associated with medication management, in addition to the fact that this professional is responsible for many other activities inherent to the specification of their profession. In addition, the involvement of non-healthcare professionals without appropriate training poses a risk to resident safety through incorrect medication use, potentially leading to errors, adverse reactions, and hospitalisations.

Future research could also focus on polypharmacy within Portuguese LTCFs, the methods employed for medication disposal, the implementation of electronic systems, and the reporting of adverse drug reactions. These topics are crucial for effective medication management and to provide valuable insights into current practices within LTCFs. Pharmacists, given their expertise, are particularly well-suited to address these areas and are essential contributors to improving medication safety and efficiency in these facilities.

Therefore, more research is imperative to define and establish the role of pharmacists in LTCFs and assess the impact of their involvement in these institutions. With this preliminary study, it is clear that there is a need to rethink the legislation regarding the healthcare team and the allocation of medication tasks in LTCFs in Portugal.

## 5. Conclusions

Population ageing is a global issue that requires countries to reorganize their social and health systems to cater to the needs of older adults. LTCFs are specifically built for individuals aged 65 and older, with a focus on independent living and active ageing. Unlike hospitals or other healthcare institutions, LTCFs are social facilities, and regulations concerning medication management are lacking.

This study emphasizes problems in medication management within Portuguese LTCFs, primarily due to the oversight by the social sector instead of the health sector. Relying on non-healthcare professionals for tasks, like medication storage, preparation, verification, and administration, poses risks to resident safety, potentially leading to medication errors and adverse reactions.

Pharmacist-led interventions in medication management are well-documented in the literature and have been proven effective in reducing medication-related problems. In the European Union, few clinical pharmacists are included in the various levels of care, such as nursing homes, emergency departments, or ambulatory interventions

The limited involvement of pharmacists in Portuguese LTCFs differs from practices in other countries, where pharmacists significantly improve medication safety and efficacy with their expertise.

The objective of this study was to evaluate medication management practices in Portuguese LTCFs and to identify the specific responsibilities of professionals involved in each task. Nurses assume a significant responsibility in the domain of medication management. However, personnel lacking the requisite training, such as assistants and auxiliary employees, are responsible for activities, such as medication preparation, validation, and administration, which could potentially jeopardize patient safety.

Further research is needed to explore the benefits of integrating pharmacist services in LTCFs and assess their impact on medication management and resident care.

As the needs of older adults increase, it is crucial to consider different perspectives in healthcare, and integrating pharmacists among LTCFs staff can be one such perspective. Consultant pharmacists could help LTCFs identify potential medication errors, provide monthly chart reviews of medications, and train non-health professionals in the LTCFs.

## Figures and Tables

**Table 1:** Demographic details of LTCFs that participated in the study and national data available in “Carta Social”.

Districts	Respondents (*n* = 392)	National (*n* = 2552)
	*n* (%)	*n* (%)
Aveiro	40 (10.20%)	137 (5.37%)
Beja	14 (3.57%)	68 (2.66%)
Braga	24 (6.12%)	164 (6.43%)
Bragança	14 (3.57%)	102 (4.00%)
Castelo Branco	17 (4.34%)	107 (4.19%)
Coimbra	18 (4.59%)	146 (5.72%)
Évora	16 (4.08%)	99 (3.88%)
Faro	11 (2.81%)	84 (3.29%)
Guarda	17 (4.34%)	143 (5.60%)
Leiria	26 (6.63%)	162 (6.35%)
Lisboa	65 (16.58%)	404 (15.83%)
Portalegre	14 (3.57%)	84 (3.29%)
Porto	34 (8.67%)	227 (8.89%)
Santarém	23 (5.87%)	171 (6.70%)
Setúbal	18 (4.59%)	145 (5.68%)
Viana do Castelo	11 (2.81%)	65 (2.55%)
Vila Real	11 (2.81%)	91 (3.57%)
Viseu	19 (4.85%)	153 (6.00%)
**Bed Capacity**		
<20	57 (14.54%)	495 (19.40%)
20–49	206 (52.55%)	1326 (51.96%)
50–99	116 (29.59%)	638 (25.00%)
>100	13 (3.32%)	93 (3.64%)
**Mean**	43.01	40.53
**Type of Ownership**		
Private	98 (25.00%)	778 (30.49%)
Social/Solidarity Institution	290 (73.98%)	1715 (67.20%)
Others	4 (1.02%)	59 (2.31%)

**Table 2:** LTCF’s staff.

Professional Categories	N. of Facilities
Technical Director	375
Sociocultural Animator, Social Educator or Geriatrics Technician	275
Physician	183
Nurse	270
Pharmacist	13
Physiotherapist	118
Nutritionist	68
Assistant	291
Person Responsible for Domestic Services	139
Cook	265
Cook’s Assistant	237
Auxiliary Employee	250
Social Worker	15
Psychologist	26
Administrative Staff	17
Other (accountants, administrators, food engineers, receptionists and drivers)	72

**Table 3:** Acquisition of medication.

	LTCF *n* (%)	Resident/Family *n* (%)	Both *n* (%)	Total *n* (%)
**Responsible for medication acquisition:**				
	206 (52.55%)	22 (5.61%)	164 (41.84%)	
**Same pharmacy?**				
Yes	177 (85.92%)	-	104 (63.41%)	281 (75.95%)
No	29 (14.08%)	-	60 (36.56%)	89 (24.05%)
**Individualised Patient Medication Packaging?**				
Yes	134 (65.05%)	-	80 (48.78%)	214 (57.84%)
No	72 (34.95%)	-	84 (51.22%)	156 (42.16%)
**Revision/Reconciliation of Medication Services?**				
Yes	201 (97.57%)	-	161 (98.17%)	362 (97.84%)
No	5 (2.43%)	-	3 (1.83%)	8 (2.16%)

**Table 4:** Medication review/reconciliation providers.

Who Provides Revision/Reconciliation of Medication?	
Institution	*n* (%)
LTCF	348 (96.13%)
Pharmacy	14 (3.87%)

**Table 5:** Storage and preservation of medications in LTCF.

Responsible for Verifying Expiration Dates and Ensuring the Proper Conditions for the Storage and Preservation of Medications.	
Institution	*n* (%)
LTCF	288 (73.47%)
Pharmacy	104 (26.53%)

**Table 6:** Storage of medications in LTCF.

Institution’s Area	Medication Without Special Storage	Refrigerated Medication	Psychoactive and Narcotic Medication
	*n* (%)	*n* (%)	*n* (%)
Dining Area	6 (1.53%)	17 (4.34%)	2 (0.51%)
Kitchen	0 (0%)	11 (2.81%)	0 (0%)
Kitchen Pantry	1 (0.26%)	8 (2.04%)	0 (0%)
LTCF Medication Storage (Internal “Pharmacy”)	41 (10.46%)	25 (6.38%)	28 (7.14%)
Medication Cart	7 (1.79%)	0 (0%)	1 (0.26%)
Medication Cabinet	9 (2.30%)	0 (0%)	23 (5.87%)
No Storage	1 (0.26%)	1 (0.26%)	1 (0.26%)
NR	10 (2.55%)	8 (2.04%)	4 (1.02%)
Nursing Office	313 (79.85%)	311 (79.34%)	330 (84.18%)
Office	1 (0.26%)	1 (0.26%)	1 (0.26%)
Resident’s Room	2 (0.51%)	0 (0%)	0 (0%)
Recreation and Activities Room	0 (0%)	1 (0.26%)	0 (0%)
Support Room	1 (0.26%)	9 (2.30%)	2 (0.51%)

**Table 7:** Verification of prepared medication.

Who Verifies the Prepared Medication?	
Other Staff Member *n* (%)	The Professional Who Prepared *n* (%)
144 (36.73%)	248 (63.27%)

## Data Availability

The data presented in this study are available on request from the corresponding author due to the presence of sensitive information which, although anonymised, may identify the institutions.

## References

[1] Eurostat Ageing Europe—Statistics on Population Developments. https://ec.europa.eu/eurostat/statistics-explained/index.php?title=Ageing_Europe_-_statistics_on_population_developments.

[2] (2022). Eurostat Healthy Life Years at Birth. https://ec.europa.eu/eurostat/statistics-explained/index.php?title=Healthy_life_years_statistics#Healthy_life_years_at_birth.

[3] Eurostat Life Expectancy at Birth by Sex. https://ec.europa.eu/eurostat/databrowser/view/tps00205/default/table?lang=en.

[4] Dericioglu D., Methven L., Clegg M.E. (2024). Understanding Age-Related Changes: Exploring the Interplay of Protein Intake, Physical Activity, and Appetite in the Ageing Population. Proc. Nutr. Soc..

[5] Lopes D.G., Mendonça N., Henriques A.R., Branco J., Canhão H., Rodrigues A.M. (2023). Trajectories and Determinants of Ageing in Portugal: Insights from EpiDoC, a Nationwide Population-Based Cohort. BMC Public Health.

[6] Mahmood M.N., Dhakal S.P. (2023). Ageing Population and Society: A Scientometric Analysis. Qual. Quant..

[7] World Health Organization Healthy Ageing and Functional Ability. https://www.who.int/news-room/questions-and-answers/item/healthy-ageing-and-functional-ability.

[8] Stypi’nska J.S., Franke A. (2023). AI Revolution in Healthcare and Medicine and the (Re-)Emergence of Inequalities and Disadvantages for Ageing Population. Front. Sociol..

[9] Lee S.W.H., Mak V.S.L., Tang Y.W. (2019). Pharmacist Services in Nursing Homes: A Systematic Review and Meta-Analysis. Br. J. Clin. Pharmacol..

[10] Dovjak P. (2022). Polypharmacy in Elderly People. Wien. Med. Wochenschr..

[11] Allin S., Martin E., Rudoler D., Church Carson M., Grudniewicz A., Jopling S., Strumpf E. (2021). Comparing Public Policies Impacting Prescribing and Medication Management in Primary Care in Two Canadian Provinces. Health Policy.

[12] Ma W., Wang H., Wen Z., Liu L., Zhang X. (2023). Potentially Inappropriate Medication and Frailty in Older Adults: A Systematic Review and Meta-Analysis. Arch. Gerontol. Geriatr..

[13] Masnoon N., Shakib S., Kalisch-Ellett L., Caughey G.E. (2017). What Is Polypharmacy? A Systematic Review of Definitions. BMC Geriatr..

[14] Pazan F., Wehling M. (2021). Polypharmacy in Older Adults: A Narrative Review of Definitions, Epidemiology and Consequences. Eur. Geriatr. Med..

[15] Fick D.M., Semla T.P., Steinman M., Beizer J., Brandt N., Dombrowski R., DuBeau C.E., Pezzullo L., Epplin J.J., Flanagan N. (2019). American Geriatrics Society 2019 Updated AGS Beers Criteria^®^ for Potentially Inappropriate Medication Use in Older Adults. J. Am. Geriatr. Soc..

[16] Khodyakov D., Ochoa A., Olivieri-Mui B.L., Bouwmeester C., Zarowitz B.J., Patel M., Ching D., Briesacher B. (2017). Screening Tool of Older Person’s Prescriptions/Screening Tools to Alert Doctors to Right Treatment Medication Criteria Modified for U.S. Nursing Home Setting. J. Am. Geriatr. Soc..

[17] Tian F., Chen Z., Zeng Y., Feng Q., Chen X. (2023). Prevalence of Use of Potentially Inappropriate Medications among Older Adults Worldwide: A Systematic Review and Meta-Analysis. JAMA Netw. Open.

[18] Roh E., Cota E., Lee J.P., Madievsky R., Eskildsen M.A. (2022). Polypharmacy in Nursing Homes. Clin. Geriatr. Med..

[19] Kim L.D., Koncilja K., Nielsen C. (2018). Medication Management in Older Adults. Cleve. Clin. J. Med..

[20] Liau S.J., Lalic S., Sluggett J.K., Cesari M., Onder G., Vetrano D.L., Morin L., Hartikainen S., Hamina A., Johnell K. (2021). Medication Management in Frail Older People: Consensus Principles for Clinical Practice, Research, and Education. J. Am. Med. Dir. Assoc..

[21] Livet M., Blanchard C., Frail C., Sorensen T., McClurg M.R. (2020). Ensuring Effective Implementation: A Fidelity Assessment System for Comprehensive Medication Management. JACCP J. Am. Coll. Clin. Pharm..

[22] Odberg K.R., Hansen B.S., Aase K., Wangensteen S. (2018). Medication Administration and Interruptions in Nursing Homes: A Qualitative Observational Study. J. Clin. Nurs..

[23] Grissinger M. (2007). Medication Errors in Long-Term Care: Part 1. Consult. Pharm..

[24] Carayon P., Wetterneck T.B., Cartmill R., Blosky M.A., Brown R., Kim R., Kukreja S., Johnson M., Paris B., Wood K.E. (2014). Characterising the Complexity of Medication Safety Using a Human Factors Approach: An Observational Study in Two Intensive Care Units. BMJ Qual. Saf..

[25] Fuller A.E.C., Guirguis L.M., Sadowski C.A., Makowsky M.J. (2018). Electronic Medication Administration Records in Long-Term Care Facilities: A Scoping Review. J. Am. Geriatr. Soc..

[26] Szczepura A., Wild D., Nelson S. (2011). Medication Administration Errors for Older People in Long-Term Residential Care. BMC Geriatr..

[27] Statistics Portugal Number of Bed in Public Hospitals. https://www.ine.pt/xportal/xmain?xpid=INE&xpgid=ine_indicadores&indOcorrCod=0008111&contexto=bd&selTab=tab2&xlang=pt.

[28] Portuguese Government Carta Social. https://www.cartasocial.pt/inicio.

[29] Pierson S., Hansen R., Greene S., Williams C., Akers R., Jonsson M., Carey T. (2007). Preventing Medication Errors in Long-Term Care: Results and Evaluation of a Large Scale Web-Based Error Reporting System. Qual. Saf. Health Care.

[30] Bitter K., Pehe C., Krüger M., Heuer G., Quinke R., Jaehde U. (2019). Pharmacist-Led Medication Reviews for Geriatric Residents in German Long-Term Care Facilities. BMC Geriatr..

[31] Kwak A., Lee E., Oh J.M., Ji E., Kim K. (2019). Perspectives of Non-Pharmacy Professionals in Long-Term Care Facilities on Pharmacist-Involved Medication Management in South Korea: A Qualitative Study. Int. J. Environ. Res. Public Health.

[32] Sadowski C.A., Charrois T.L., Sehn E., Chatterley T., Kim S. (2020). The Role and Impact of the Pharmacist in Long-Term Care Settings: A Systematic Review. J. Am. Pharm. Assoc..

[33] Erzkamp S., Rose O. (2018). Development and Evaluation of an Algorithm-Based Tool for Medication Management in Nursing Homes: The AMBER Study Protocol. BMJ Open.

[34] Bužančić I., Kummer I., Držaić M., Ortner Hadžiabdić M. (2022). Community-Based Pharmacists’ Role in Deprescribing: A Systematic Review. Br. J. Clin. Pharmacol..

[35] Gooen L.G. (2017). Medication Reconciliation in Long-Term Care and Assisted Living Facilities: Opportunity for Pharmacists to Minimize Risks Associated with Transitions of Care. Clin. Geriatr. Med..

[36] Elliott R.A., Tran T., Taylor S.E., Harvey P.A., Belfrage M.K., Jennings R.J., Marriott J.L. (2012). Gaps in Continuity of Medication Management during the Transition from Hospital to Residential Care: An Observational Study (MedGap Study). Australas. J. Ageing.

[37] Chia H.S., Ho J.A.H., Lim B.D. (2015). Pharmacist Review and Its Impact on Singapore Nursing Homes. Singap. Med. J..

[38] Verrue C., Mehuys E., Boussery K., Adriaens E., Remon J.P., Petrovic M. (2012). A Pharmacist-Conducted Medication Review in Nursing Home Residents: Impact on the Appropriateness of Prescribing. Acta Clin. Belg..

[39] Lee C.Y., Beanland C., Goeman D., Petrie N., Petrie B., Vise F., Gray J., Elliott R.A. (2018). Improving Medication Safety for Home Nursing Clients: A Prospective Observational Study of a Novel Clinical Pharmacy Service—The Visiting Pharmacist (ViP) Study. J. Clin. Pharm. Ther..

[40] Leguelinel-Blache G., Castelli C., Rolain J., Bouvet S., Chkair S., Kabani S., Jalabert B., Rouvière S., Choukroun C., Richard H. (2020). Impact of Pharmacist-Led Multidisciplinary Medication Review on the Safety and Medication Cost of the Elderly People Living in a Nursing Home: A before-after Study. Expert. Rev. Pharmacoecon Outcomes Res..

[41] Armistead L.T., Hughes T.D., Larson C.K., Busby-Whitehead J., Ferreri S.P. (2021). Integrating Targeted Consultant Pharmacists into a New Collaborative Care Model to Reduce the Risk of Falls in Older Adults Owing to the Overuse of Opioids and Benzodiazepines. J. Am. Pharm. Assoc..

[42] Hiu S., Tam Y., Hirsch J.D., Watanabe J.H. (2017). Medication Regimen Complexity in Long-Term Care Facilities and Adverse Drug Events-Related Hospitalizations. Consult. Pharm..

[43] Levenson S.A., Saffel D.A. (2007). The Consultant Pharmacist and the Physician in the Nursing Home: Roles, Relationships, and a Recipe for Success. J. Am. Med. Dir. Assoc..

[44] The United States Government Get Started with Medicare. https://www.medicare.gov/basics/get-started-with-medicare.

[45] The United States Government Medication Therapy Management Programs for Complex Health Needs. https://www.medicare.gov/drug-coverage-part-d/what-medicare-part-d-drug-plans-cover/medication-therapy-management-programs-for-complex-health-needs.

[46] O’shea T.E., Zarowitz B.J., Erwin W.G. (2017). Comprehensive Medication Reviews in Long-Term Care Facilities: History of Process Implementation and 2015 Results. J. Manag. Care. Spec. Pharm..

[47] Garland C.T., Guénette L., Kröger E., Carmichael P.H., Rouleau R., Sirois C. (2021). A New Care Model Reduces Polypharmacy and Potentially Inappropriate Medications in Long-Term Care. J. Am. Med. Dir. Assoc..

[48] Stuhec M. (2021). Clinical Pharmacist Consultant in Primary Care Settings in Slovenia Focused on Elderly Patients on Polypharmacy: Successful National Program from Development to Reimbursement. Int. J. Clin. Pharm..

[49] Stewart D., Whittlesea C., Dhital R., Newbould L., McCambridge J. (2020). Community Pharmacist Led Medication Reviews in the UK: A Scoping Review of the Medicines Use Review and the New Medicine Service Literatures. Res. Soc. Adm. Pharm..

[50] World Health Organization (2016). Global Strategy on Human Resources for Health: Workforce 2030.

[51] Walpola R.L., Issakhany D., Gisev N., Hopkins R.E. (2024). The Accessibility of Pharmacist Prescribing and Impacts on Medicines Access: A Systematic Review. Res. Soc. Adm. Pharm..

[52] MacDonald O., Smith K., Marven M., Broughton N., Geddes J., Cipriani A. (2020). How Pharmacist Prescribers Can Help Meet the Mental Health Consequences of COVID-19. Evid. Based Ment. Health.

[53] Weeks G., George J., Maclure K., Stewart D. (2016). Non-Medical Prescribing versus Medical Prescribing for Acute and Chronic Disease Management in Primary and Secondary Care. Cochrane Database Syst. Rev..

[54] Piraux A., Bonnan D., Ramond-Roquin A., Faure S. (2024). The Community Pharmacist as an Independent Prescriber: A Scoping Review. J. Am. Pharm. Assoc..

[55] Dawoud D., Griffiths P., Maben J., Goodyer L., Greene R. (2011). Pharmacist Supplementary Prescribing: A Step toward More Independence?. Res. Soc. Adm. Pharm..

[56] Dennis M., Haines A., Johnson M., Soggee J., Tong S., Parsons R., Sunderland B., Czarniak P. (2022). Cross-Sectional Census Survey of Patients with Cancer Who Received a Pharmacist Consultation in a Pharmacist Led Anti-Cancer Clinic. J. Cancer Educ..

[57] Soggee J., Hunt M., O’Callaghan B., Lam W.S., Cannell P., Boardman G., Sunderland B., Czarniak P. (2023). Specialist Pharmacist Consultations with Cancer Patients in a Pharmacist-Led Anticancer Clinic. Asia Pac. J. Clin. Oncol..

[58] Firkus D., McCoy R.G., Matulis J., Kessler M., Mara K., Herges J. (2023). Evaluation of Pharmacist Consults within a Collaborative Enhanced Primary Care Team Model to Improve Diabetes Care. PLoS ONE.

[59] Portuguese Government Legislação Aplicável Às Respostas Sociais. https://www.cartasocial.pt/legislacao-aplicavel-as-respostas-sociais.

[60] De C., Bonfim J., Saraiva M.E. Centro de Dia. *Guiões Técnicos* 1996. https://www.seg-social.pt/documents/10152/13328/Centro_dia/f8de1cb2-a6e8-4137-8a7f-4d76233e58bc/f8de1cb2-a6e8-4137-8a7f-4d76233e58bc.

[61] Portuguese Government Portaria n.^o^ 96/2013, 4 de Março. https://diariodarepublica.pt/dr/detalhe/portaria/96-2013-259261.

[62] Portuguese Government Portaria n.^o^ 38/2013, 30 de Janeiro. https://diariodarepublica.pt/dr/detalhe/portaria/38-2013-258278.

[63] Portuguese Government Decreto-Lei n.^o^ 391/91, de 10 de Outubro. https://diariodarepublica.pt/dr/detalhe/decreto-lei/391-1991-288067.

[64] Portuguese Government Portaria n.^o^ 349/2023, 13 de Novembro. https://diariodarepublica.pt/dr/detalhe/portaria/349-2023-224139049.

[65] Portuguese Government Declaração de Retificação n.^o^ 1/2024. https://diariodarepublica.pt/dr/detalhe/declaracao-retificacao/1-2024-836495390.

[66] Kalideen L., Govender P., van Wyk J.M. (2022). Standards and Quality of Care for Older Persons in Long Term Care Facilities: A Scoping Review. BMC Geriatr..

[67] Morin L., Laroche M.L., Texier G., Johnell K. (2016). Prevalence of Potentially Inappropriate Medication Use in Older Adults Living in Nursing Homes: A Systematic Review. J. Am. Med. Dir. Assoc..

[68] Tommelein E., Mehuys E., Petrovic M., Somers A., Colin P., Boussery K. (2015). Potentially Inappropriate Prescribing in Community-Dwelling Older People across Europe: A Systematic Literature Review. Eur. J. Clin. Pharmacol..

[69] O’Dwyer M., Peklar J., Mccallion P., Mccarron M., Henman M.C. (2016). Factors Associated with Polypharmacy and Excessive Polypharmacy in Older People with Intellectual Disability Differ from the General Population: A Cross-Sectional Observational Nationwide Study. BMJ Open.

[70] Anrys P.M.S., Strauven G.C., Foulon V., Degryse J.M., Henrard S., Spinewine A. (2018). Potentially Inappropriate Prescribing in Belgian Nursing Homes: Prevalence and Associated Factors. J. Am. Med. Dir. Assoc..

[71] Portuguese Government (2007). Decreto-Lei n.o 307/2007, 31 de Agosto.

[72] Rodrigues A.R., Teixeira-Lemos E., Mascarenhas-Melo F., Lemos L.P., Bell V. (2022). Pharmacist Intervention in Portuguese Older Adult Care. Healthcare.

[73] da Saúde D.-G. (2016). *Norma n.^o^ 018/2016*; Lisbon, Portugal. https://normas.dgs.min-saude.pt/wp-content/uploads/2019/10/reconciliacao-da-medicacao.pdf.

[74] Świtalski J., Wnuk K., Tatara T., Miazga W., Wiśniewska E., Banaś T., Partyka O., Karakiewicz-Krawczyk K., Jurczak J., Kaczmarski M. (2022). Interventions to Increase Patient Safety in Long-Term Care Facilities—Umbrella Review. Int. J. Environ. Res. Public Health.

[75] Kapoor A., Field T., Handler S., Fisher K., Saphirak C., Crawford S., Fouayzi H., Johnson F., Spenard A., Zhang N. (2019). Adverse Events in Long-Term Care Residents Transitioning from Hospital Back to Nursing Home. JAMA Intern. Med..

[76] Hyttinen V., Jyrkkä J., Valtonen H. (2016). A Systematic Review of the Impact of Potentially Inappropriate Medication on Health Care Utilization and Costs Among Older Adults. Med. Care.

[77] Portuguese Government Ministry of Labour, Solidarity, and Social Security. https://www.portugal.gov.pt/pt/gc24/area-de-governo/trabalho-solidariedade-e-seguranca-social/acerca.

[78] Portuguese Government (2006). Decreto-Lei n.o 101/2006, 6 de Junho.

[79] Lopes H., Mateus C., Hernández-Quevedo C. (2018). Ten Years after the Creation of the Portuguese National Network for Long-Term Care in 2006: Achievements and Challenges. Health Policy.

[80] Ordem dos Farmacêuticos (2018). Manual de Boas Práticas de Farmácia Hospitalar.

[81] Ordem dos Farmacêuticos (2009). Manual de Boas Práticas Farmacêuticas Para a Farmácia Comunitária.

[82] Portuguese Government (2021). Deliberação n.o 946/2021.

[83] Carolo D., Estevão P., Santi J. Impacto Da COVID-19 Nos Lares de Idosos; Estudos CoLABOR, Ed.; 2023. https://static1.squarespace.com/static/63fcc2d5a2b083728c24708d/t/649c73bd429cc76445dc4d83/1687974846827/estudos_6.pdf.

[84] Grupo de Trabalho Colaborativo Referencial de Boas Práticas Na Prestação de Cuidados de Saúde Nas ERPIs; 2022. https://www.igas.min-saude.pt/wp-content/uploads/2023/05/ERPI_Referencial_de_Boas_Praticas_VFinal.pdf.

[85] Dilles T., Elseviers M.M., Van Rompaey B., Van Bortel L.M., Stichele R.R.V. (2011). Barriers for Nurses to Safe Medication Management in Nursing Homes. J. Nurs. Scholarsh..

[86] Murry L.T., Murry R.C., Deng H., Viyyuri B., Gerleman B.L., Urmie J. (2021). Community Pharmacy Medicare Part D Consultations: Plan-Switching Decisions and Chronic Medication Adherence. J. Pharm. Pract..

[87] Murry L.T., Witry M.J., Urmie J.M. (2023). A Qualitative Exploration of Patient Preferences for Medicare Part D Consultation Services Offered in a Community Pharmacy Setting. J. Am. Pharm. Assoc..

[88] Steinman M.A. (2019). Reducing Hospital Admissions for Adverse Drug Events through Coordinated Pharmacist Care: Learning from Hawai’i without a Field Trip. BMJ Qual. Saf..

[89] Entsuah N., Early N., Hanson L., Brucato B., Fairman K.A., Naberhaus T. (2022). Outcomes of Pharmacist-Conducted Admission Medication-Regimen Reviews in Long-Term Care Facilities. Sr. Care Pharm..

